# Supplementary material for: Controlling gene activation by enhancers through a drug-inducible topological insulator
Source: eLife. 2020 May 5;9:e47980. doi: 10.7554/eLife.47980 (PMC7200164; doi:10.7554/eLife.47980)
Supplement: Transparent reporting form [file elife-47980-transrepform.docx]

***eLife’s* transparent reporting form**

We encourage authors to provide detailed information *within their submission* to facilitate the interpretation and replication of experiments. Authors can upload supporting documentation to indicate the use of appropriate reporting guidelines for health-related research (see [EQUATOR Network](http://www.equator-network.org/%20)), life science research (see the [BioSharing Information Resource](https://biosharing.org/)), or the [ARRIVE guidelines](http://www.plosbiology.org/article/info:doi/10.1371/journal.pbio.1000412) for reporting work involving animal research. Where applicable, authors should refer to any relevant reporting standards documents in this form.

If you have any questions, please consult our Journal Policies and/or contact us: [editorial@elifesciences.org](mailto:editorial@elifesciences.org).

**Sample-size estimation**

- You should state whether an appropriate sample size was computed when the study was being designed
- You should state the statistical method of sample size computation and any required assumptions
- If no explicit power analysis was used, you should describe how you decided what sample (replicate) size (number) to use

Please outline where this information can be found within the submission (e.g., sections or figure legends), or explain why this information doesn’t apply to your submission:

We did not perform sample-size estimation. We used sample sizes that are commonly used for each type of experiments.

**Replicates**

- You should report how often each experiment was performed
- You should include a definition of biological versus technical replication
- The data obtained should be provided and sufficient information should be provided to indicate the number of independent biological and/or technical replicates
- If you encountered any outliers, you should describe how these were handled
- Criteria for exclusion/inclusion of data should be clearly stated
- High-throughput sequence data should be uploaded before submission, with a private link for reviewers provided (these are available from both GEO and ArrayExpress)

Please outline where this information can be found within the submission (e.g., sections or figure legends), or explain why this information doesn’t apply to your submission:

The definition of replicates is described in Materials and Methods. The number of replicates is indicated either in figure legends or as individual data points in the plots. All the deep sequencing data of the 4C-seq, RNA-seq and nChIP-seq libraries analyzed in this study were deposited in ArrayExpress: E-MTAB-7668, E-MTAB-7669, E-MTAB-7670, E-MTAB-8492. The deposit of the 4C-seq data presented in Figure 8-figure supplement 2E-G is being finalized at the submission of the revised manuscript.

**Statistical reporting**

- Statistical analysis methods should be described and justified
- Raw data should be presented in figures whenever informative to do so (typically when N per group is less than 10)
- For each experiment, you should identify the statistical tests used, exact values of N, definitions of center, methods of multiple test correction, and dispersion and precision measures (e.g., mean, median, SD, SEM, confidence intervals; and, for the major substantive results, a measure of effect size (e.g., Pearson's r, Cohen's d)
- Report exact p-values wherever possible alongside the summary statistics and 95% confidence intervals. These should be reported for all key questions and not only when the p-value is less than 0.05.

Please outline where this information can be found within the submission (e.g., sections or figure legends), or explain why this information doesn’t apply to your submission:

Statistical analysis methods are described in the figure legends and in Materials and Methods. P-values are reported along the plots.

(For large datasets, or papers with a very large number of statistical tests, you may upload a single table file with tests, Ns, etc., with reference to sections in the manuscript.)

**Group allocation**

- Indicate how samples were allocated into experimental groups (in the case of clinical studies, please specify allocation to treatment method); if randomization was used, please also state if restricted randomization was applied
- Indicate if masking was used during group allocation, data collection and/or data analysis

Please outline where this information can be found within the submission (e.g., sections or figure legends), or explain why this information doesn’t apply to your submission:

Experimental groups are defined in Materials and Methods as well as in the figure legends.

**Additional data files (“source data”)**

- We encourage you to upload relevant additional data files, such as numerical data that are represented as a graph in a figure, or as a summary table
- Where provided, these should be in the most useful format, and they can be uploaded as “Source data” files linked to a main figure or table
- Include model definition files including the full list of parameters used
- Include code used for data analysis (e.g., R, MatLab)
- Avoid stating that data files are “available upon request”

Please indicate the figures or tables for which source data files have been provided:

Source data files are provided for Figure 6F and 6G, Figure 7J, Figure 8C, and Figure 8-figure supplement 1B, in which summary data, but not individual data of replicates, are plotted. We provide the source data files for the 4C-seq read counts in given intervals in Figures 1, 3, 5, 8, and Figure 8-figure supplement 2E-G. We also provide the R codes as well as the input files to perform the PCA for the 4C-seq data in Figures 3 and 8. We also provide the read counts assigned to genes of the RNA-seq data as well as the results of the DESeq2 analysis for Figure 2. We also provide the ChIP-seq count data against peaks that we used to prepare Figure 4C-E and Figure 5-figure supplement 1F.
